# When Art, Science, and Culture Commingle

**DOI:** 10.1371/journal.pbio.1000100

**Published:** 2009-05-19

**Authors:** Cheryl A. Kerfeld

**Affiliations:** 1 Structural Genomics and Education Programs, United States Department of Energy, Joint Genome Institute, Walnut Creek, California, United States of America; 2 Department of Plant and Microbial Biology, University of California, Berkeley, California, United States of America

## Abstract

Cheryl Kerfeld reviews Tactical Biopolitics, a collection of essays that reveals the constructive exchanges and “tribal skirmishes” that inevitably arise when departmentalized minds explore the boundaries of science, art, and politics.

The history of modern science is punctuated by moments when the fruits of science captivate the public imagination. Traces of these impressions can be found in works of art; for instance, one sees the influence of 17th century astronomy on poetry in *Paradise Lost*, as when Satan stops by the sun to ask for directions to the earth, Milton alludes to Galileo's discovery of sunspots: “There lands the Fiend, a spot like which perhaps/Astronomer in the Sun's lucent Orbe/Through his glaz'd Optic Tube yet never saw” and in the sudden emergence of the ellipse in baroque architecture [1]. More recently, scholars have argued for the influence of relativity theory on the development of cubist painting [2] and of both relativity and quantum mechanics on the poetry of T.S. Eliot [3]. (“What might have been is an abstraction/Remaining a perpetual possibility/Only in a world of speculation.”)[Fig pbio-1000100-g001]

**Figure pbio-1000100-g001:**
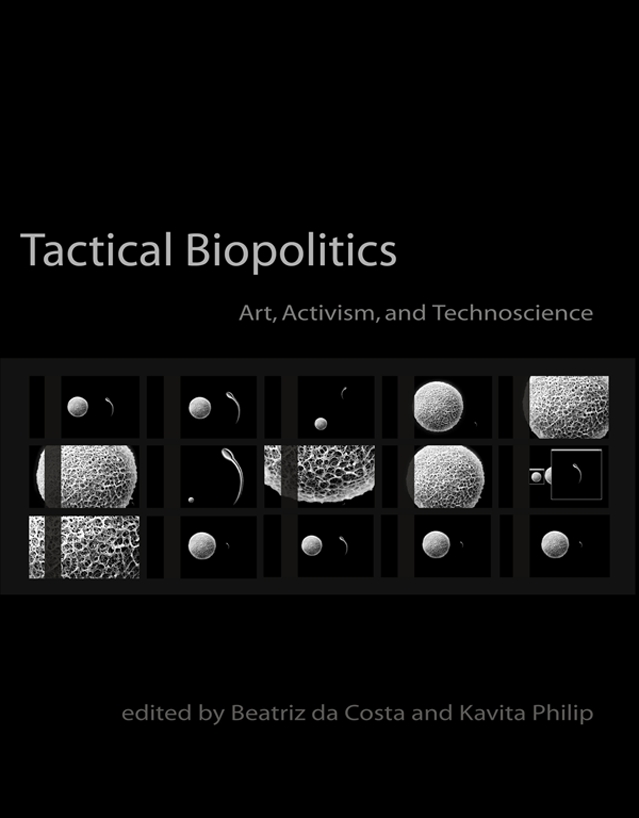
da Costa B, Philip K, editors (2008) Tactical Biopolitics: Art, Activism and Technoscience. Cambridge (Massachusetts): MIT Press. 504 p. ISBN (hardcover): 978-0262042499. US$40.00.

Whole cultural movements have been considered a response to the prevailing scientific world view as seen, for example, in the “Romantic Reaction” to the mechanized worldview of the 18th century (e.g., in the words of Schlegel, “The explanation of an organic product, of an organic being must be historical, not mechanical”[4]) [5],[6]. At the same time, the cultural climate can influence the imagination of scientists; it has been proposed that Darwin's construction of natural selection has roots in Romantic ideals [7], and the thematic similarities found in cubist painting and relativity theory merely demonstrate that both art and science are creative enterprises shaped by the preoccupations of the culture in which they are immersed [8].

Now, in the “biological century,” with the concurrent revolution in new technologies to communicate and even create new life forms, how are art, science, and culture influencing one another? *Tactical Biopolitics*, edited by Beatriz da Costa and Kavita Philip, offers one part of the answer by providing a look at how artists and other nonscientists are inspired and provoked by contemporary biological research.

The premise of *Tactical Biopolitics* is “that the political challenges at the intersection of life science and art are best addressed through a combination of artistic intervention, critical theorizing and reflective practices.” The term Tactical Biopolitics “is a creative terminological misappropriation, drawing its inspiration from, but not directly mapping onto, two formations: the assembly of resistant cultural practices referred to as *Tactical Media*, and the intellectual ferment around the history of biopolitics.” Tactical Media has been described as do-it-yourself media activism that is “never impartial,” and Biopolitics situates these activities in the historical framework of Foucault's concept of biopower, in which biotic factors are manipulated to regulate society.

*Tactical Biopolitics,* then, is a collection of essays organized by themes—Curating the Book of Life, The Biolab and the Public, Gendered Science, Expertise and Amateur Science, Biosecurity and Bioethics—that capture both the constructive exchanges and the tribal skirmishes that take place when life, science, art, and politics meet. It is also a record of “the possible recuperation of one of [Tactical Media's] strongest aspects: the inter- and ‘(un)-disciplinary’ exchanges among practitioners and theorists from various backgrounds, always privileging collaboration and coordination with larger strategy-based movements of resistance to hegemonic forces….we now call for the inclusion and cooperation of the scientific community.”

To understand this interdisciplinary exchange, one must be prepared to respect local idiomatic customs. A biologist exploring *Tactical Biopolitics* encounters strange semantic flora and fauna—unfamiliar juxtapositions, and novel fusions of adjectives and nouns–that make it unmistakable that this is an alternative domain for the life sciences. At their best, the unusual verbal combinations invite contemplation; others set up an impenetrable language barrier or read a little like poetry: “An immeasurable amount of productive energy is wasted appeasing the anxiety inserted by capital through insidious and invasive manipulations of huge sections of the public imaginary.” And, as in any good interdisciplinary conversation, readers get asked questions they would never have thought to ask themselves: “How can we know for sure these days that the truck driver repairing his exhaust at the crossroads in your neighborhood is not a silent conceptual artist engaging you in a thought-through performative experience?”

How indeed? Such questions appear throughout the text and in different formulations of what is and how to be a “biological citizen.” The responses come from a phenotypically diverse range of nonscientists: artists, various disciplinary theorists, and activists. Critical theorist Claire Pentecost describes the role of the artist in terms of puncturing the barrier between “science under neo-liberalism” and “an alienated public.” She also finds similarities between scientists and artists here: “In some obvious ways, artists face many of the same challenges scientists do in relation to an alienated public. Blockbuster museum shows apart, contemporary ‘fine art’ is a small, misunderstood subculture.” There are other accounts by and about artists and artworks—cue the GFP bunny Alba, the albino rabbit genetically altered with green fluorescent protein. The artist Eduardo Kac pushed the boundaries of whether it is socially acceptable for artists to create transgenic animals. In *Tactical Biopolitics*, the story is told with an emphasis on what happened after the French laboratory that created Alba refused to release her to the artist (who planned to live with the glowing animal in a museum). This turned out to be a serendipitous departure from the artist's experimental plan by generating a flood of publicity and an ongoing debate about whether life should be manipulated for art's sake. Other essays document the experiences of artists, activists, and members of the public, in the laboratories from where they report their impressions, such as, “the scientific laboratory may be just an overelaborate kitchen designed by scientists to mystify the sciences behind closed doors”

In contrast, essays on biofiction show it to be a potent force for demystifying science and cultivating interdisciplinary understanding. Sci-Fi novelist Gwyneth Jones recounts seeking out a scientist who would allow her into the laboratory to develop a novel. The partnership succeeded, Jones says, because Jane Davies, a developmental geneticist, could “recognize and nurture what faint resonance it had with her professional knowledge,” and the novel *Life* was born. Jones feels lucky that Davies “grasped the idea of a *doubled* narrative, where the information, the sequence of events, is meant to convey at least two meanings at the same time.” But on other further reflection, she glimpses common ground under science with its models and metaphors and storytelling. “Or perhaps that wasn't luck. The genome is the original complex layered, looping, interactive narrative.”

By packaging scientific concepts in flesh and blood, fiction can be a useful means of conveying the scientific worldview. (It has likewise been suggested to be useful for doing philosophy: “If you want to be a philosopher, write novels” [9].) Fictional narratives can be seen as thought experiments that “can raise important questions without necessarily answering or resolving them,” write Karen Cardozo and Banu Subramaniam in the essay *Genes, Genera and Genres.* The pair reviews the novel *All Over Creation*, which simultaneously uses multiple layers of metaphors to explore, for example, nature and nurture through the effects of the environment on the development of potatoes and on the self actualization of daughters, and on the physical and metaphysical development of hybrid plants and people, both at “ground zero for self-inflicted bioterrorism.” It is the special province of fiction to slowly develop plots that work on multiple levels and both entertain and enlighten. Cardozo and Subramaniam's deft interpretation of *All Over Creation* makes one wish for more novels like it in which fiction is used to embody a deep structure of scientific concepts that could be dissected out in a public forum like Oprah's book club.

Cardozo and Subramaniam are an interdisciplinary team—one trained in biology, the other literary studies—and their *naturcultures* approach (Donna Haraway's term) epitomizes the synergy of interdisciplinary exploration. In contrast, others roll into the intersection of the life sciences and art in the intellectual equivalent of a Humvee. In *Biotech Patronage*, Jacqueline Stevens, a political scientist, decodes the influence of corporate America on the iconography of recent public art–science exhibitions. In one of several examples, she critiques an installation created by a collaboration of artists and scientists (some are both), called *Ecce Homology*, that alluded to the similarities between the human and the rice genome. (The installation was encapsulated, not unlike a nucleus, within a cultural history museum that also contained a show called “The Art of Rice” and “From the Verandah” [10].) The installation translated the amino acid sequences of human and rice carbohydrate catabolism genes into pictographs projected onto the axes of the gallery wall. The viewer could select one by motioning to it and, through subsequent fragmentation and reassembly, the pictograph would be matched to its counterpart in the other genome. It was a wall-sized metaphor for BLAST. As in her other case studies, Stevens insinuates that the creators were appeasing their corporate sponsor, because BLAST…“requires exactly the high-speed computing technologies sold by the exhibit's main funder, Intel.” This is a hypothesis untroubled by testing—Stevens hasn't tried to check the facts. (In the interests of full disclosure: I was part of the group who created *Ecce Homology*; we sought out Intel's sponsorship after coming up with the concept, their support enabled us to realize our vision. At the time I was asked to review *Tactical Biopolitics*, neither I, nor the editors of *PLoS Biology* knew that *Ecce Homology* was discussed in the book (it isn't in the index).)

Nor is Stevens favorably impressed by the interdisciplinary result. “Despite the low quality of science education in this country, it is more likely that a student would be able to notice flaws in the exhibit's presentation of scientific data than its inaccurate claim to roots in Nietzsche,” she writes. “The pun ‘Ecce Homology’ plays on the fact that *homo*- is from a Greek root that means ‘earth’ and means ‘man’ (in contrast with gods); and *hom*- is from a Greek root meaning ‘same.’ To understand this, one would have to look for history, meaning, and difference, all of which BLAST destroys.” The wry allusion of *Ecce Homology*, which was overlooked by Stevens, was to Nietzsche's use of “ecce homo” in *Twilight of the Idols* (“Let us finally consider how naïve it is altogether to say ‘Man *ought* to be such and such!’ Reality shows us an enchanting wealth of types, the abundance of a lavish play and change of forms—and some wretched loafer of a moralist comments: ‘No! Man ought to be different.’ He even knows what man should be like, this wretched bigot and prig: he paints himself on the wall and comments, ‘*Ecce homo*!’” [11]) and his late reflection on his own work [12], *Ecce Homo.* Instead, according to Stevens, “their invocation of Nietzsche might be classified as a crime against philosophy.” The lesson from this essay for scientists is that an amateur interest in humanism (unlike an amateur interest in science?) is not always welcome. It can still be productively count among a biologist's leisure pursuits; think of Darwin passing time on The Beagle reading *Paradise Lost* with his subconscious absorbing the garden imagery and the supernatural selection process set in motion by the *felix culpa*.

Highly departmentalized minds patrolling the borders of their disciplines come with interdisciplinary territory. One of the limitations of old media, such as a book of essays, is that one can read each piece as a monolog rather than as an opening gambit in an ongoing conversation. But new media, tactical or not, will undoubtedly increase the exchange of perspectives and foster new collaborative thought experiments and creations of art and fiction—and science. *Tactical Biopolitics* is a snapshot of the state-of-the-art at one of the farthest frontiers of interdisciplinary exploration.
